# Complete Mitochondrial DNA Diversity in Iranians

**DOI:** 10.1371/journal.pone.0080673

**Published:** 2013-11-14

**Authors:** Miroslava Derenko, Boris Malyarchuk, Ardeshir Bahmanimehr, Galina Denisova, Maria Perkova, Shirin Farjadian, Levon Yepiskoposyan

**Affiliations:** 1 Institute of Biological Problems of the North, Russian Academy of Sciences, Magadan, Russia; 2 Institute of Molecular Biology, National Academy of Sciences of Armenia, Yerevan, Armenia; 3 Immunology Department, Shiraz University of Medical Sciences, Shiraz, Iran; University of Florence, Italy

## Abstract

Due to its pivotal geographical location and proximity to transcontinental migratory routes, Iran has played a key role in subsequent migrations, both prehistoric and historic, between Africa, Asia and Europe. To shed light on the genetic structure of the Iranian population as well as on the expansion patterns and population movements which affected this region, the complete mitochondrial genomes of 352 Iranians were obtained. All Iranian populations studied here exhibit similarly high diversity values comparable to the other groups from the Caucasus, Anatolia and Europe. The results of AMOVA and MDS analyses did not associate any regional and/or linguistic group of populations in the Anatolia/Caucasus and Iran region pointing to close genetic positions of Persians and Qashqais to each other and to Armenians, and Azeris from Iran to Georgians. By reconstructing the complete mtDNA phylogeny of haplogroups R2, N3, U1, U3, U5a1g, U7, H13, HV2, HV12, M5a and C5c we have found a previously unexplored genetic connection between the studied Iranian populations and the Arabian Peninsula, India, Near East and Europe, likely the result of both ancient and recent gene flow. Our results for Persians and Qashqais point to a continuous increase of the population sizes from ∼24 kya to the present, although the phase between 14-24 kya is thought to be hyperarid according to the Gulf Oasis model. Since this would have affected hunter-gatherer ranges and mobility patterns and forced them to increasingly rely on coastal resources, this transition can explain the human expansion across the Persian Gulf region.

## Introduction

Due to its geo-strategic location, the Middle East has served as a key crossroad for human dispersals and played a critical role in the migrations between the populations of the Middle East and beyond [1], [2]. The most important long-term factor in this process was human adaptation to the region’s geographical, topographical and climatic conditions with the subsequent development of agriculture, pastoralism, and pastoral nomadism. Variety of the people that populate the area is often affected by the regional geography: while certain geographic features, such as the Dasht-e Kavir and Dasht-e Lut deserts in Iran and the Hindu Kush mountains in eastern Afghanistan have served as potential barriers [2]–[6], others, such as the Strait of Bab el Mandab and the region along the southern coast of Iran, Afghanistan and Pakistan known as Baluchestan, have acted as conduits for human dispersals [2], [7], [8]. Furthermore, environmental fluctuations occurring over time have changed areas that once served as a passageway to a barrier, as in case of the Strait of Hormuz that connects the Arabian Peninsula to present day Iran [9].

Although Paleolithic and Mesolithic people left their mark in the Iranian Plateau, major human population developments with possible genetic implications occurred here during the Neolithic period and later [1], [10], [11]. The Middle Eastern region spanning from Zagros Mountains and northern Mesopotamia to Southeast Anatolia, called Fertile Crescent, is broadly accepted to be the place where agriculture first arose [1]. Important agricultural developments occurred in the eastern horn of the Fertile Crescent, notably in Elam (southwestern Iran), connecting Mesopotamia and the Iranian Plateau [12]. The highly urban Elamite civilization had close contacts with Mesopotamians but exhibited an extensive differentiation from the rest of the Fertile Crescent populations, including a language that is thought to belong to the Dravidian family [3], [13]. Another major innovation, that most likely emerged later than agriculture, was the domestication of animals, which is thought to have led to dramatic population expansions in Eurasia [1], [14], [15]. Starting about 5000 years (ky) before present, pastoral nomadism developed in the grasslands of Central Asia, as well as in southeastern Europe, opening up the possibility of rapid movements of large population groups [16]. The spread of these new technologies has been associated with the dispersal of Dravidian and Indo-European languages in southern Asia [17], [18]. It is hypothesized that the proto-Elamo-Dravidian language, most likely originated in the Elam province in southwestern Iran, spread eastwards with the movement of farmers to the Indus Valley and the Indian sub-continent [13], [19].

Between the third and second millennia BCE the Iranian Plateau became exposed to incursions of pastoral nomads from the Central Asian steppes, who brought the Indo-Iranian language of the Indo-European family, which eventually replaced Dravidian languages, perhaps by an elite-dominance model [13], [17], [20].

Already at the beginning of the first millennium BCE the population of the Iranian Plateau consisted of agriculturalists and pastoralists representing a variety of ethnic groups. In the mid-sixth century BCE onward, the unification of Mesopotamian lowlands and the Zagros highlands resulted in the creation of several successive highland world empires (Achaemenid, Parthian, and Sassanid) that lasted, with a brief Greek interruption, for more than 1 ky [12]. These empires would dominate part of the Middle East until the Islamic expansion in the region in the seventh century CE. In the period of the seventh to thirteenth centuries CE the Arab-Muslim, Seljuq and subsequent Turkic-Mongol invasions signaled the arrival of new peoples with certain flocks and cultures. Specifically, in a series of rapid Arab-Muslim conquests in the seventh century, the Arab armies swept through most of the Middle East, completely engulfing the Persian lands [21]. The dominance of the Arabs came to a sudden end in the mid-eleventh century with the arrival of Seljuq Turks, originating from the Oghuz tribes. The expanding waves of these Altaic-speaking nomads from Central Asia involved regions farther to the west, such as Iran, Iraq, Anatolia, and the Caucasus, where they imposed Turkic languages [22]. Later, the Mongol armies also moved westward and, by the early thirteenth century, established their rule over a vast region, including Iran and advancing as far west as the Caucasus and Turkey [1], [21]. These waves of various invasions and subsequent migrations resulted in major demographic expansions in the region, which added new languages and cultures to the mix of peoples that had pre-existed in Iran.

With the objective of gaining a comprehensive understanding of the impact that complex historical migrations and events have had upon the genetic structure of populations, mitochondrial DNA (mtDNA) analysis has often proven to be a highly effective tool [23], [24]. Previous examinations of the maternal gene pools of Iranians have revealed a genetic connection between Iranian populations and the Indian sub-continent and the Arabian Peninsula, likely the result of both ancient and recent gene flow. Furthermore, the regional distribution of certain mtDNA haplogroups provides evidence of barriers to gene flow posed by the two major Iranian deserts and the Zagros mountain range [11], [25], [26]. The contention that these geographical barriers may have restricted genetic flow within Iran and between Iran and neighboring regions is further supported by Y-chromosome data [2], [4], [27]. Besides, a discordant pattern of high ethno-linguistic and low mtDNA heterogeneity was observed for the comprehensive set of Iranian populations, which can be partly explained by both geographical factors and cultural/linguistic differences acting as barriers to matrilineal gene flow [28].

It should be noted, however, that the above-mentioned matrilineal studies are hindered by their utilization of the limited set of mtDNA markers (control-region sequence data combined with RFLP analysis of coding region markers) that severely restricted their ability to define phylogeographic patterns and perform molecular dating correctly. Though some set of complete mtDNA genomes from the Near East and adjacent territories has been published recently, the Iranian mtDNAs were under-represented there [29]–[39]. To date, only one study dealing with complete mtDNA variation in Iranians has been published [40], but the small sample size renders the resulting sequences unsuitable for comprehensive phylogenetic and demographic analyses.

In order to shed light on the genetic structure of the Iranian population as well as on the expansion patterns and population movements which affected this region, we present here a large-scale complete mtDNA analysis of 352 Iranians, representative of the majority of the provinces and ethnic groups, with a special attention to the three major ethnic groups, i.e. Indo-European-speaking Persians and Turkic-speaking Qashqais and Azeris.

## Materials and Methods

### Ethics Statement

The study was approved by the Ethics Committee of the Institute of Biological Problems of the North, Russian Academy of Sciences, Magadan, Russia (statement no. 002/012 from 15 March, 2012). All subjects provided written informed consent for the collection of samples and subsequent analysis.

### Sample collection and analysis of mtDNA sequence variation

Sampling localities are shown in Figure S1. The sample consisted of 352 unrelated individuals from 25 Iranian provinces and belonging to 13 different ethnic groups (in parentheses): 2 from Ardabil (Azeris), 13 from Isfahan (9 Persians, 4 Armenians), 8 from Khuzestan (6 Persians, 1 Bakhtiari, 1 Armenian), 6 from Mazandaran (3 Mazandaranis, 2 Persians, 1 Gilak), 1 from Alborz (Persian), 2 from Bushehr (Persians), 15 from East Azarbaijan (14 Azeris, 1 Armenian), 1 from West Azerbaijan (Azeri), 119 from Fars (9 Persians, 110 Qashqais), 7 from Gilan (5 Persians, 1 Azeri, 1 Gilak), 5 from Golestan (3 Persians, 2 Turkmens), 1 from Hamadan (Khalaj), 1 from Ilam (Lur), 101 from Kerman (Persians), 4 from Kermanshah (3 Persians, 1 Kurd), 2 from Kohgiluyeh and Boyer-Ahmad (Lurs), 1 from Kurdistan (Kurd), 4 from Luristan (2 Lurs, 1 Persian, 1 Bakhtiari), 9 from Markazi (7 Persians, 1 Armenian, 1 Khalaj), 2 from North Khorasan (1 Persian, 1 Khorosani), 3 from Qazvin (Persians), 15 from Razavi Khorasan (3 Khorosani, 1 Kurd, 11 Persians), 1 from Sistan and Baluchestan (Persian), 27 from Tehran (1 Mazandarani, 3 Armenians, 3 Azeris, 1 Indian, 19 Persians), 1 from Yazd (Persian), and 1 from Zanjan (Azeri). Detailed information concerning geographic origin of parents and grandparents was obtained from all donors (see Table S1, for details on the geographic origin of the donors).

Genomic DNA from whole blood was extracted using a standard phenol/chlorophorm procedure. Saliva samples were processed using Oragene ® DNA Collection Kits (DNA Genotek, Canada), following the instructions of the manufacturer. We performed the complete mtDNA sequencing as previously described [41] using an ABI 3500xL Genetic Analyzer. DNA sequence data were analyzed using SeqScape 2.5 software (Applied Biosystems) and compared to the revised Cambridge reference sequence (rCRS) [42]. A nomenclature, which we hereby update, follows [43] with several new modifications.

### Statistical analysis and molecular dating

The most-parsimonious trees of the complete mtDNA sequences were reconstructed manually, and verified by means of the Network 4.5.1.0 software [44] and mtPhyl software (http://eltsov.org). The time to the most recent common ancestor (TMRCA) for each cluster was calculated by computing the averaged distance (ρ) of all the haplotypes in a clade to the respective root haplotype [45]. Heuristic estimates of the standard error (s) were calculated from an estimate of the genealogy [46]. Calculations were obtained using the entire mtDNA genomes but excluding hot spot mutations such as 16182C, 16183C, and 16519. Values of mutation rates based on mtDNA complete genome variability data and synonymous substitutions [47] were used.

In order to detect population growth, we obtained the Bayesian skyline plots (BSPs) [48] from BEAST 1.6.1 [49] for the Iranian complete mtDNA sequences with a relaxed molecular clock and the HKY model of nucleotide substitutions with gamma distributed rates as in [50]. Each MCMC sample was based on a run of 60000000 generations sampled every 6000 steps, with the first 6000000 generations regarded as burn-in. Three independent runs were made for each set of sequences, and a mutation rate of 1.665×10^−8^ [47] was used. We checked for convergence to the stationary distribution and sufficient sampling by inspection of posterior samples. We generated BSPs for each population and some sub-clades of mtDNA tree and visualized the plots with Tracer v1.4.

DnaSP 5.10.01 [51] was used to calculate the basic parameters of genetic diversity. The Analysis of molecular variance (AMOVA) was carried out using Arlequin 3.5.1.2 [52]. The statistical significance of Fst values was estimated by permutation analysis, using 10000 permutations. The STATISTICA 10 (StatSoft Inc., Tulsa, OK, USA) was used for multi-dimensional scaling (MDS) analysis [53]. Published population data on complete mtDNA variability in Azeris, Armenians and Georgians from Caucasus, Turks and Iranians from western Asia [40], Sardinians from eastern Sardinia [54] and Tatars from the Volga-Ural region [55] were included in our comparative analysis.

The GenBank (http://www.ncbi.nlm.nih.gov/genbank) accession numbers for the 355 novel complete mtDNA sequences (352 Iranian mtDNAs and 3 Russian H13 mtDNAs) reported in this paper are KC911275-KC911629.

## Results

### Population summary statistics, population relationships and demographic analysis

Summary statistics describing genetic diversity in Iranian populations are shown in Table 1. Overall diversity is very high, with 315 different haplotypes found among 352 individuals. A total of 1267 polymorphic sites were detected in combined Iranian data set. The number of mean pairwise differences is 33.94, ranged from 33.67 in Qashqais to 35.24 in Persians. All groups exhibit similar haplotype and nucleotide diversity values, as well as an excess of low-frequency variants that is characteristic of a recent population expansion, as shown by significantly negative values for Tajima’s D. Levels of genetic variation in Iranian populations are comparable to the other groups from the Caucasus, Anatolia and Europe (Table S2).

**Table 1:** Diversity indices and neutrality test values for Iranian populations based on complete mtDNA sequences.

Population	No. of samples	No. of haplotypes, h	No. of variable sites, S	Haplotype diversity, Hd (S.D.)	Nucleotide diversity, Pi (S.D.)	Average number of nucleotide differences, k	Tajima’s D
Iranians (Total)	352	315	1267	0.999 (0.000)	0.00208 (0.0004)	33.94	−2.58 (P < 0.001)
Persians	181	164	913	0.999 (0.001)	0.00213 (0.00006)	35.24	−2.54 (P < 0.001)
Qashqais	112	94	617	0.996 (0.002)	0.00204 (0.00007)	33.67	−2.4 (P < 0.01)
Azeris	22	22	225	1.000 (0.014)	0.00212 (0.00014)	34.54	−1.81 (P < 0.05)

**Figure 1:**
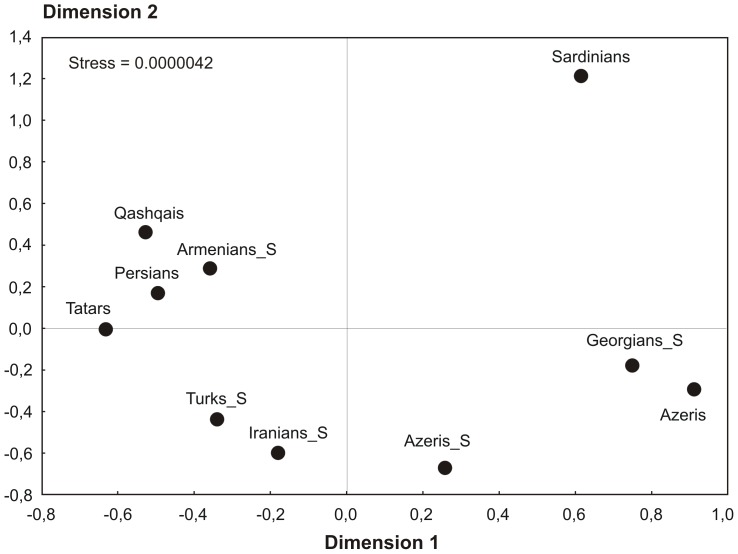
MDS plot based on Fst statistics calculated from complete mtDNA sequences for population samples from Iran, Anatolia, Caucasus, and Europe. The populations from [40] labeled with “S” after underscore.

In order to visualize the relationships between Iranians studied and other populations of the Caucasus, Anatolia and Europe based on complete mtDNA sequence data, MDS plot was constructed from the pairwise Fst values (Table S3). The results show that Persians and Qashqais are close to each other and to Armenians, whereas Azeris from Iran are located nearby Georgians. It is worth pointing out the position of Azeris from the Caucasus region, who despite their supposed common origin with Iranian Azeris, cluster quite separately and occupy an intermediate position between the Azeris/Georgians and Turks/Iranians grouping (Figure 1). Interestingly, the results of our MDS analysis do not combine the populations studied according to their geographic and/or linguistic affinity. Therefore, Turkic-speaking Qashqais, Azeris, and Turks are located quite distantly from each other on the plot, even though association between the latter two groups has been recently revealed based on complete mtDNA sequences [40]. All populations from the Caucasus region (Armenians, Azeris, and Georgians) are scattered on the plot though their genetic proximity has been demonstrated by Schönberg et al. [40]. Similarly, Iranians from Tehran province [40] and Persians studied here are clearly separated from each other.

The genetic structure of Iranians in comparison with the populations from the Caucasus, Anatolia and Europe was investigated by AMOVA (Table S4). As expected, before grouping, the majority of variability was due to within population component (98.5% for Iranian populations only and 98.12% for the complete data set). After grouping, neither geographic nor linguistic classification gave a good fit to the genetic data. A slightly higher degree of geographic rather than linguistic correlation with the genetic structuring of the examined populations emerges only when the geographically and genetically distant population of Buryats from Inner Mongolia region of China was added to the studied data set, thus underlining the importance of geographic distance. It should be noted that correlation between geographical proximity and genetic relationships of populations has been shown previously based on HVS1 variability data for Indo-European and Semitic-speaking groups of southwestern Iran [56], [57]. Moreover, a slightly better fit of geographic rather than linguistic classification of populations to the complete mtDNA sequence data has been demonstrated recently for Iranian, Anatolian and Caucasus region populations [40].

**Figure 2:**
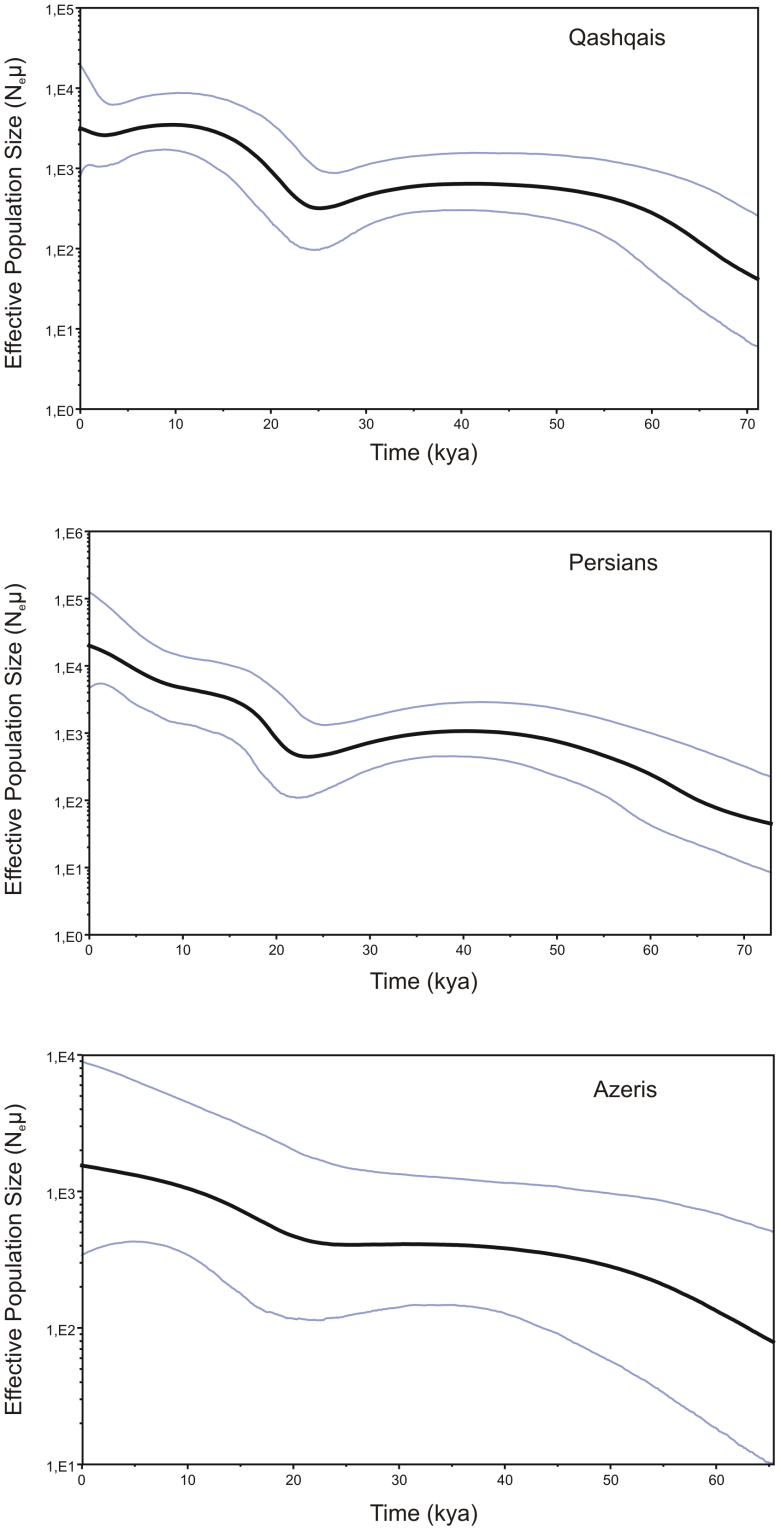
BSP indicating the median of the hypothetical effective population size through time based on complete mtDNA genome data for Persians, Qashqais and Azeris. Maximum time (x axis) corresponds to the median posterior estimate of the genealogy root-height.

The BSPs obtained for Persians and Qashqais are generally similar, pointing to a first population expansion around 40–42 kya, followed by a gradual decrease of population size up to ∼ 24 kya. The BSP for Persians show a continuous, slightly stepped increase of population size to the present, whereas the BSP for the Qashqais data separates two steps (∼10 kya and ∼2.5 kya). The BSP for the Azeris has different pattern, pointing only to a period of gradual increase from ∼27 kya (Figure 2).

### The topology of Iranian mtDNA tree and haplogroup profile distribution

The haplogroup assignment for each individual according to the nomenclature of Phylotree.org [43] (Build 15) is given in Table S4. A total of 212 different sub-haplogroups or paragroups (unclassified lineages within a clade) were identified, which fall into 75 principal haplogroups. The vast majority of the mtDNAs clustered into macrohaplogroups M, N, and R, but a limited number was found to belong to the sub-Saharan haplogroups L2a, L3d, L3e, L3f and L5c (Figure S2). Two haplogroups, H91 and HV18, are defined here for the first time, whereas others (marked red in Table S1 and Figure S2) represent newly identified sub-clades. Moreover, for some previously known haplogroups we redefined diagnostic markers that allow a better definition of the haplogroup topology within the tree.

Haplogroup frequencies for Azeris, Persians, Qashqais and the entire Iranian mtDNA data set are presented in Table 2. All Iranian populations studied here are characterized by the same most prominent western Eurasian mtDNA haplogroups, H, J, T and U; however, the frequency distribution of these lineages varies between different populations. All the three populations show similar frequencies of haplogroup U (22.7%, 24.3%, and 22.3%, respectively), but the frequencies of haplogroups H and J are more pronounced in Qashqais (28.6% and 15.2%, respectively) than in Persians (16.6% and 6.1%, respectively) and Azeris (22.7% and 4.6%, respectively). In contrast, haplogroup T dominates in Azeris (18.2%) and Persians (11.6%), being found only in 4.5% in Qashqais. Another notable difference between Azeris and two other populations studied is the higher frequency of haplogroups X2 (9.1% versus 2.8% in Persians and 2.7% in Qashqais) and N1a3 (9.1% versus 0.6% in Persians), and the absence of haplogroups U and H diversification, which may be merely the result of smaller sample size. There are also distinguished differences between the Persians and Qashqais with respect to the distribution of some sub-haplogroups, including H13 (2.8% versus 6.3%), T2 (7.2% versus 1.8%), U3 (2.8% versus 8%), and U7 (7.2% versus 2.7%) (Table 2).

**Table 2:** Mitochondrial haplogroup frequencies (%) in Iranian populations.

Haplogroup	Azeris (N = 22)	Persians (N = 181)	Qashqais (N = 112)	Iranians (Total)(N = 352)
A4	0	0	0.89	0.57
B4	0	0	0	0.28
C4	4.55	0	1.79	0.85
C5	0	0.55	0	0.57
D4	0	0.55	1.79	1.14
F1b1	0	0	0	0.28
G2a3	4.55	0	0	0.28
R0	0	2.21	1.79	1.7
HV*	0	2.76	0	1.42
HV1	0	1.1	0.89	1.14
HV2	0	3.31	0.89	1.99
HV5	0	0	0	0.28
HV9	0	0.55	0	0.28
HV12	0	0.55	1.79	1.14
HV13	0	1.1	0	0.57
HV14	0	0.55	0	0.28
HV16	0	0	2.68	0.85
HV18	0	1.1	0	0.85
V	0	0.55	0	0.28
H*	0	2.21	4.46	3.13
H1*	0	3.31	0	1.7
H1e1a5	0	0	4.46	1.42
H2a	0	0.55	0.89	0.85
H3	0	1.1	0	0.57
H5	4.55	1.66	0	1.14
H6a1	0	0	0	0.28
H7	0	0.55	1.79	0.85
H10	0	0.55	0	0.28
H13	0	2.76	6.25	3.98
H14	0	1.1	0.89	0.85
H15	4.55	0.55	3.57	1.7
H18b	0	0	3.57	1.14
H20	4.55	0	0	0.28
H29	4.55	0	0	0.28
H49	4.55	0	0	0.28
H57	0	1.1	0	0.57
H63	0	0.55	0	0.28
H66	0	0.55	0	0.28
H91	0	0	2.68	0.85
H (Total)	22.7	16.6	28.6	20.7
R2	0	3.31	2.68	2.56
J1b	0	4.97	7.14	6.53
J1c	0	1.1	3.57	1.7
J1d	4.55	0	4.46	1.7
J (Total)	4.55	6.08	15.2	9.94
T1	0	4.42	2.68	3.41
T2	18.2	7.18	1.79	5.97
T (Total)	18.2	11.6	4.46	9.38
R5	0	0.55	0	0.28
R8	0	0	0.89	0.28
R30	0	1.1	0	0.57
U1	9.09	2.76	1.79	3.13
U2	4.55	2.21	0.89	1.99
U3	0	2.76	8.04	3.98
U4	0	3.31	2.68	2.84
U5	0	2.21	3.57	2.56
U7	0	7.18	2.68	5.4
U8	0	0.55	0.89	0.57
K	9.09	3.31	1.79	3.69
U (Total)	22.7	24.31	22.32	24.15
I1	0	1.1	0	0.85
I2	4.55	0	0	0.28
I (Total)	4.55	1.1	0	1.14
N1a3	9.09	0.55	0	0.85
N1b1	0	0.55	0	0.28
N2a	0	1.66	0	0.85
W	0	4.97	5.36	4.55
N3	0	1.11	1.79	1.14
X2	9.09	2.76	2.68	2.84
M2	0	0.55	0	0.28
M3	0	0.55	0	0.28
M4	0	0.55	0.89	0.57
M5	0	3.87	0	1.99
M18	0	0.55	0	0.28
M42	0	0.55	0	0.28
L2a	0	1.11	0	0.57
L3d	0	0	1.79	0.57
L3e	0	0.55	0	0.28
L3f	0	0	0.89	0.28
L5c	0	0.55	0	0.28
L (Total)	0	2.21	2.68	1.99

All three Iranian populations studied here exhibit similar frequencies of western Eurasian component, represented by the haplogroups N1, N2, N3, X, R0, R2’JT, and U, accounting for 90.9% in Azeris, 86.7% in Persians and 91.1% in Qashqais. The eastern Eurasian lineages, represented by haplogroups A4, B4, C4, C5, D4, F1b1, G2a3, account for 9.1% of mtDNAs in Azeris, 1.1% - in Persians, and 4.5% - in Qashqais. This is consistent with the data presented by Quintana-Murci et al. [11] showing absence or low frequencies of eastern Eurasian haplogroups in the populations from the Anatolian/Caucasus region and the Iranian Plateau. The South Asian influence mainly represented by haplogroups M2, M3, M4, M5, M18, M42, R5, R8, R30, and the two sister clades U2c and U2d are more pronounced in Persians (9.9%) than in Qashqais (1.8%). As expected, all Indian-specific mtDNAs in Iran originate from the southern provinces of the country (Table S1). The same observation is true for the African-specific lineages, represented by haplogroups L2a, L3d, L3e, L3f and L5c, found in Persians and Qashqais with similar frequencies of 2.2% and 2.7%, respectively. These findings coincide with the data of Terreros et al. [26] who reported the same proportion of African L haplotypes (2.6%) for the southern region of Iran, but are in contrast with the data published by Quintana-Murci et al. [11], where L lineages are reported among the northern but not the southern groups of Iran.

### Phylogeography of specific haplogroups

**Figure 3:**
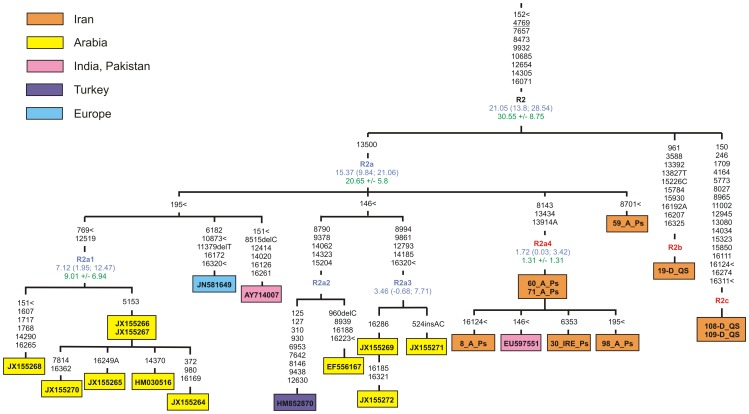
Maximum-parsimony phylogenetic tree of complete mtDNA sequences belonging to haplogroup R2. Numbers along links refer to substitutions scored relative to rCRS [42]. Transversions are further specified; ins and del denote insertions and deletions of nucleotides, respectively; back mutations are underlined; symbol < denotes parallel mutation. Iranian samples labeled as in Table S1, for published data the accession number in indicated. The box containing the sample ID is color coded according to the geographic origin of the sample. Time estimates (in kya) shown along links next to clade labels are based on the complete mtDNA genome clock (marked in blue) and the synonymous clock (marked in green) [47]. Established haplogroup labels are shown in black; blue are redefined and red are newly identified haplogroups in the present study.

It is known that some of the mtDNA lineages are either autochthonous to Iran or underwent a major expansion in this region [11]. Among them is haplogroup R2, which is concentrated in southern Pakistan and in India, and is present at low frequencies in the most of adjacent regions, including the Near East, the Caucasus, the Iranian Plateau, the Arabian Peninsula, and Central Asia [11], [25], [37]. The extensive sequencing of complete mtDNAs from a large part of the Iranian Plateau led us to the identification of several highly divergent Qashqai lineages within the entire haplogroup R2 and revealed a new Persian-specific sub-clade within haplogroup R2a. The reconstructed complete mtDNA phylogeny based on fifteen published and nine new Iranian R2 sequences is shown in Figure 3 and includes age estimates obtained from complete genome and synonymous mutations. As can be seen, haplogroup R2 has a likely pre-LGM/Late Glacial time depth, characterized by an overall coalescence time estimate of 21–31 kya, and it probably originated in the southern region of Iran and split early into three branches. The first sub-clade, R2a, dates to ∼15–20 kya and includes several branches found mostly in southern Arabians (R2a1, R2a2, R2a3) and Persians (R2a4), as well as single mtDNAs from South Asia, the Near East and Europe. Two other mtDNA lineages, which are named here as R2b and R2c, restricted to Qashqais from southern Iran and potentially could have split before the main R2a sub-clade (sample size does not allow dating).

Although U7 is a typical Near Eastern and Indian haplogroup [11], [25], its phylogeny is still poorly resolved, mainly due to the paucity of complete mtDNA genome sequence data. Here, we present the reconstructed phylogeny of haplogroup U7 based on 44 complete mtDNA genomes including nineteen newly sequenced samples from Iran (Figure S3). The complete mtDNA sequences form two distinct clades, both with considerable internal variation and different geographic distribution. They were termed U7a and U7b according to the established mtDNA tree [43] thus modifying an earlier proposal [58]. One of the fully sequenced Indian U7 mtDNAs (C22 from Palanichamy et al. [59] does not belong to either U7a or U7b and represents probably a novel lineage which we call here U7c.

U7a is the most diverse clade of U7 and it is found in virtually every population where the U7 haplogroup has been identified and sampled. The phylogeny of U7a reveals at least five sub-clades (with similar coalescence time estimates varying from 9 to 20 kya), which are most frequent in India, Iran and the Near East. Interestingly, a large subset of Iranian U7a samples could not be ascribed to any of its known sub-clades thus showing that more sampling of Iranian and Indian populations is highly desirable to uncover the ample diversity of this haplogroup. The second main sub-clade, U7b, dates to ∼6–11 kya and includes several mtDNAs found predominantly in Europe (Figure S3), indicating a likely evolution *in situ*.

We tested our set of complete mtDNA sequences for expansion signal(s) using BSP (Figure S4). For U7, the initial expansion seems to more or less coincide with the ∼16–22 kya estimated coalescent age for the entire U7 and ∼19–21 kya for the most diverse and prevalent sub-clade U7a. This expansion appears to have continued at a somewhat equal rate, gradually slowing down, until the curve even drops slightly, and eventually a new expansion phase takes place around ∼4 kya.

Besides haplogroup U7, sub-haplogroups U1 and U3 exist at considerable levels in the Iranian populations studied, reaching their highest frequencies in Azeris (9%) and Qashqais (8%), respectively (Table 2). To further assess the variability of haplogroups U1 and U3 found in the mitochondrial gene pool of Iranians we reconstructed the complete mtDNA genome phylogeny based on our and all available published data (Figure S3). It is obvious that these haplogroups have a likely pre-LGM time depth characterized by an overall coalescence time estimates of 38–50 kya and 32–41 kya, respectively, and both have a very distinctive geographic distribution which might be highly informative about the demographic history of the Middle East. Haplogroup U1 presents two basal branches, named - U1a’c and U1b. The latter is found mostly among Europeans, and its estimated age of ∼9–14 kya indicates a postglacial or Late Glacial expansion. Sub-clade U1a’c, with coalescence age estimate of 29–44 kya, is the most represented of U1 clades, and it probably originated in Southwest Asia and split early into three branches. The first branch, U1a, comprises a series of sub-clades (U1a1, U1a2, U1a3, and U1a4) dating to 13–15 kya; it was found across Southwest and South Asia, the Caucasus region and Europe, but at least one lineage within U1a3, which we called here U1a3b, was restricted to Iran. It is characterized by coalescence age estimates of about 10–16 kya thus placing its origin to postglacial or Late Glacial time. It should be noted, that the Persian-specific U1a3a branch is also found in Sardinians, and its estimated age of 8–10 kya points to a long-standing link between them.

Like haplogroup U1, haplogroup U3 falls into two distinct sub-clades, U3a’c and U3b, with almost the same coalescence age estimated as 18–26 kya and 18–24 kya, respectively (Figure S3). U3a’c divides into the major U3a, found in Europe, the Near East, the Caucasus and northern Africa, and the minor U3c sub-clade, represented by a single Azeri mtDNA from the Caucasus. U3b is also widespread across the Middle East and the Caucasus, and it is found especially in Iran, Iraq and Yemen, with a minor European sub-clade, U3b1b, dated to ∼2–3 kya. The other almost-entirely European sub-clade, U3a1, dates to ∼4–7 kya, suggesting a relatively recent (late Holocene or later) expansion of these lineages in Europe.

**Figure 4:**
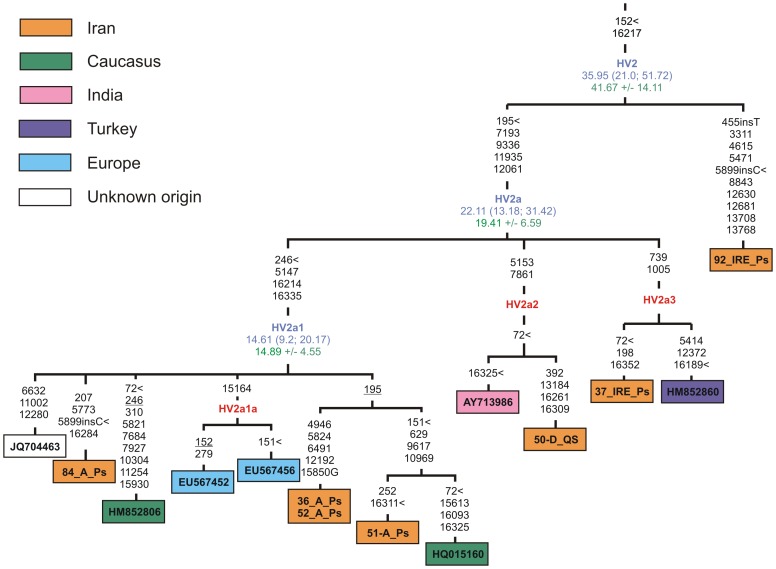
Maximum-parsimony phylogenetic tree of complete mtDNA sequences belonging to haplogroup HV2. Designations are as in Figure 3.

In the current study we have reconstructed the phylogeny of haplogroup HV2 based on fourteen complete mtDNA genomes including seven newly sequenced Iranian samples and the revised classification of this haplogroup that was defined earlier as having one main branch – HV2a [43]. The addition of our Persian sequence (92_IRE_Ps) to the tree gives a branching point for the entire HV2, now defined by two control region transitions at nps 152 and 16217 (Figure 4). Noteworthy, the addition of a substantial set of completely sequenced mtDNAs from Iranian populations has allowed us to reveal at least three different sub-clusters within the HV2a haplogroup, HV2a1, HV2a2, and HV2a3. The main sub-clade, HV2a1, dates to ∼15 kya and includes several branches from the Caucasus, Europe and Iran, whereas the sub-clade HV2a2 shows more limited distribution being found only in Iran and India. As a whole, the HV2a sub-clade dates to the Late Glacial period (19–22 kya), whereas the deeper HV2 clade shows the coalescence age estimates of 36–42 kya, closely matching with the timeframe of modern human arrival in the Near East [60]. The observation of earliest diverging sequences in the HV2 complete genome tree prior to the emergence of HV2a in Iran, might suggest a possible Iranian origin for haplogroup HV2.

**Figure 5:**
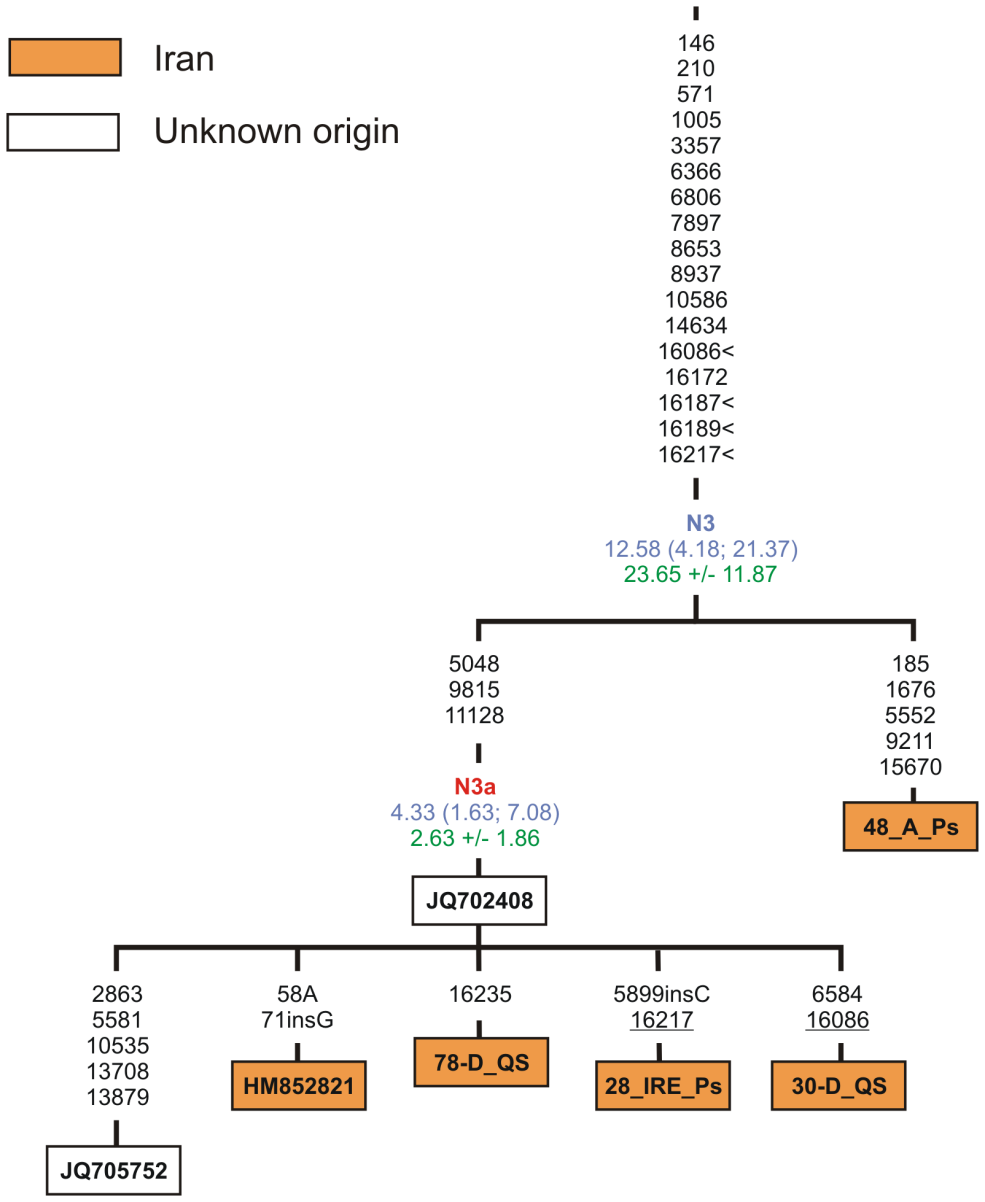
Maximum-parsimony phylogenetic tree of complete mtDNA sequences belonging to haplogroup N3. Designations are as in Figure 3.

Another mtDNA sub-clade, N3, most likely evolved exclusively within Iran. Despite the coalescent age estimate for the N3a branch, combining the majority of N3 mtDNAs found so far, is about of 3–4 kya only, the finding of Iranian sample (48_A_Ps) sharing 17 out of 20 polymorphisms with N3a, indicates a deeper, close to the LGM time, split between these Iranian-specific lineages (Figure 5).

Another haplogroup frequent in the Iranian populations is H13. It encompasses 16% and 21.9% of Persian and Qashqai haplogroup H samples, respectively, which makes its total frequency in the Iranian haplogroup H gene pool 19.2% - the highest rate reported to date. The complete mtDNA phylogeny of haplogroup H13 based on all available published data and our newly sequenced fourteen Iranian and three Russian mtDNAs is presented in Figure S3. As shown, H13 has a likely LGM time depth characterized by an overall coalescence time estimate of 20–24 kya. Three of its major sub-clades, H13a, H13b, and H13c, have roughly similar ages of 17–23 kya, 16–19 kya and 17–24 kya, respectively. Haplogroup H13a, the most represented of H13 clades, is further subdivided into two principal sub-clades, H13a1 and H13a2, each containing several independent branches. Some of these sub-clades have very distinctive geographic distribution, though the large set of H13 samples from [61] is of unspecified descent. Whilst the European and Caucasian lineages occur throughout the tree, the overwhelming majority of Iranian H13 mtDNAs clustered into sub-group H13a2 with a coalescence age estimated set as 14–16 kya. Notably, inside haplogroup H13a2a at least two novel subgroups, H13a2a1 and H13a2a2, specific to the Iranian populations have been revealed. H13a2a1 is found solely in Qashqais, and its estimated age of ∼2.5 kya indicates a recent founder effect among Qashqais ancestors. The second sub-clade, H13a2a2, dates to 12–16 kya and includes several branches characteristic of Persians, Qashqais and Indians. Thus, a relatively large amount of internal variation accumulated in the Iranian-specific branch of H13 would mean that H13a2a also might have arose *in situ* in the Iranian Plateau after the arrival of the H13a2a founder from somewhere else in the Near East/Caucasus region. The coalescence age estimates suggest that H13a2a expanded ∼12–16 kya, an expansion time that matches the continuous population increase from ∼20 kya to the present observed in the BSP obtained from the overall H13 data (Figure S4).

It should be noted, that besides H13a2a1, some other sub-haplogroups of H and T show recent founder effects ∼ 2–3 kya both in Qashqais (H1e1a5; H18b) and Persians (H1ca; T1a1m; T2i2) (Figure S2).

Traces of relatively recent gene flows from the Indian sub-continent are revealed in the Iranian mtDNA gene pool by the presence of the well resolved sub-haplogroup M5a, originated probably in central India and spread out to its eastern and western regions around 13–17 kya [62]. This lineage is observed at an overall frequency of 2% in Iran, being detected only in Persians with the frequency of 3.9% (Table 2). According to the complete mtDNA phylogeny, the majority of Iranian M5a-sequences forms a specific sub-clade, M5a2a4, dating to 3–5 kya (Figure S3).

## Discussion

Overall, the complete mtDNA sequence analysis revealed an extremely high level of genetic diversity in the Iranian populations studied which is comparable to the other groups from the South Caucasus, Anatolia and Europe. The results of AMOVA and MDS analyses did not associate any regional and/or linguistic group of populations in the Anatolia/Caucasus and Iran region pointing to strong genetic affinity of Indo-European speaking Persians and Turkic-speaking Qashqais, thus suggesting their origin from a common maternal ancestral gene pool. The pronounced influence of the South Caucasus populations on the maternal diversity of Iranian Azeris is also evident from the MDS analysis results.

The Iranian populations studied here and previously [11], [25], [26] exhibit similar mtDNA lineage composition and mainly consist of a western Eurasian component, accounting for about 90% of all samples, with a very limited contribution from eastern Eurasia, South Asia and Africa. The South Asian and African influence is more pronounced in Iranians from the southern provinces of the country.

Our results confirms that populations from Iran, Anatolia, the Caucasus and the Arabian Peninsula display a common set of maternal lineages although considerable regional differences in haplogroup frequencies exist [11], [29]. Meanwhile, some haplogroups previously defined as South Asian (such as R2 and HV2) could be considered as having Southwest Asian origin, taking into account the relatively high frequency and diversity of those haplogroups in Iran. Although R2 is a very rare haplogroup, the phylogeographic analysis indicates that it is present mostly in southern Arabia, while it has been suggested that the unrepresented Near East can be considered as a possible place of origin for R2 [37]. Meanwhile, our data indicate that haplogroup R2 has a likely pre-LGM/LGM time depth, with a coalescence time estimate of 21–31 kya, and it probably originated in southern Iran, although the neighboring Gulf Oasis region cannot be excluded, taking into account the close genetic affinity between Persians and Arabians proposed by Terreros et al. [26]. One should note also that the age estimate for the extremely rare haplogroup N3, which is specific for Iranian populations, is close to the LGM time, as it has been dated to 13–24 kya.

We observed that haplogroup HV2, dated at 36–42 kya, most likely arose in Iran between the time of the first settlement by modern humans and the LGM. The antiquity of the Indian and Southwest Asian-specific sub-clade HV2a with a coalescence age estimate at 19–22 kya allows us to suggest an Iranian ancestry for HV2a and its transfer from Iran to India in repeated gene flows from west to east, which have been, as suggested by Quintana-Murci et al. [11], more common than those from east to west. The presence of the haplogroup U7 in the Indian sub-continent also attests to the close genetic resemblance between India and Iran and may suggest gene flow between the two regions. U7 is virtually absent in western and eastern European populations and is present at low frequencies (up to 5%) in the Near East, the Caucasus, Central Asia, and the Indian sub-continent. The highest frequencies of this haplogroup are registered in some Iranian populations (up to 10%) and in Gujarat (over 12%), the westernmost state of India [11], [25], [26], [63]. The expansion times and haplotype diversities for the Indian and Near Eastern haplogroup U7 HVS1 sequences are strikingly similar, suggesting some degree of genetic continuum spanning from the Near East through northwest India and reaching north into Central Asia somewhere between 30–50 kya [25]. Moreover, the coalescence time estimates for South- and West Asian-specific sub-branches of haplogroup U7 also predate the LGM, pointing to a deep autochthonous history of this haplogroup in the region.

Here we dissect haplogroup U7 mtDNAs into three phylogenetic clusters characterizing by Southwest Asian/Indian (U7a and U7c) and European (U7b) distribution. The initial expansion for the haplogroup U7 coincides with the time at ∼16–22 kya, which is within the temporal bounds for the other autochthonous Iranian haplogroups, such as the aforementioned R2, HV2 and N3.

Haplogroup U3 is also restricted primarily to the Near East [64], with the age estimate of ∼33 kya according to [47] or 32–41 kya according to the present data, including 14 new mtDNA genomes from Iran. Two sub-clusters, U3b1a and U3b3, were highly divergent in the Near East, with the ages of 22–33 and 18–26 kya, respectively. Among them, U3b3 lineages appear to be restricted to populations of Iran and the Caucasus, while the sub-cluster U3b1a is common in the whole Near East region.

Haplogroup H is the most frequent lineage in the Near East and Europe, but the coalescent age estimates for H in the Near East are significantly older than in Europe (23–28 kya versus 19–21 kya, respectively) [64]. It has been suggested earlier that the first expansions of the haplogroup H may have taken place in the northern part of the Near East and the southern Caucasus, where the oldest clades of haplogroup H are present [31]. However, most of the Near Eastern/Caucasus and North African variants of haplogroup H started to expand after the LGM, between 18 and 10 kya [31], [65]. Certain sub-clades of haplogroup H are more prevalent in the Near East and the Caucasus (H1, H2a1, H4, H5, H6, H7, H13, H14, H15, H18, H20), and only several sub-clades (H6, H13, H14) coalesce to the pre-LGM period [31], [47], [65]–[67]. Among them, only the sub-clade H13 has been relatively frequent and divergent in the Iranian populations studied. It is known that haplogroup H13 reaches the highest frequency in the Caucasus (in Daghestan and Georgia) [31]. Although all the H13 samples in the Caucasus and in Europe fall into H13a, the largest sub-clade of H13, additional H13 lineages also are present in the southern Caucasus and Near East populations [31]. The haplogroup H13 and three of its major sub-clades, H13a, H13b, and H13c, show the coalescence age estimates lying in a range of 16–24 kya, thus placing their origin during the LGM and even before.

It should be noted that the coalescence age estimates obtained for all of the haplogroups discussed here overlap with a significant warming of the Earth’s climate occurred between 26–33 kya [68] as well as with a more humid condition in the Near East before the LGM (25–31 kya) [69]–[71], favorable for the first population expansions. In addition, Fernandes et al. [38] have demonstrated that some minor mtDNA haplogroups, such as N1, N2 and X, most likely spread from the Persian Gulf Oasis region toward the Near East and Europe during the pluvial periods dated from 24–55 kya and from 6–12 kya [72]. Moreover, BSP analysis of the past population size changes based on mtDNA diversity within haplogroups R2, R0a and HV1, representing 22% of the total mtDNA gene pool of the southern Arabia, has shown that the Arabian population underwent a large expansion already some 12–13 kya [37]. Similarly, the results obtained from the N1, N2 and X data from the Near East, Arabia and eastern Africa demonstrate a continuous increase of population size from ∼15 kya [38], consistent with the population expansion in the Levant region during the wet phase between 15 and 13 kya [73]. Our results for Persians and Qashqais point to a continuous increase of the population sizes from ∼24 kya to the present, though the phase between 14–24 kya is thought to be hyperarid in the hinterlands of the Gulf [72]. Undoubtedly, this would have affected hunter-gatherer ranges and mobility patterns due to transformation and disappearance of the interior savannas of Arabia and forced them to increasingly rely on coastal resources, because large spaces of fertile land in the Gulf basin were exposed [72]. This transition can explain the human expansion across the Persian Gulf region in accordance with the Gulf Oasis model.

Previous studies of the mtDNA HVS1 sequence variation in populations of the Near East and Europe identified back-migrations from Europe to the Near East [64]. It has been suggested that haplogroups U5 and V, which most likely evolved in Europe, have been introduced to the Near East recently. Haplogroup V is very rare in the Near Eastern populations as well as in the Iranian populations studied here, being found only once in the Persian sample. As for haplogroup U5, it is widespread, albeit at low frequencies, in different populations of the Near East [64], [74] and the majority of the Near Eastern U5 haplotypes belongs to the sub-cluster U5a1a’g defined by a back mutation at np 16192. Notably, six of eight U5a haplotypes found in Iranians also belong to U5a1a’g and four of them belong to the very rare sub-cluster U5a1g. Since U5a1g is defined by a single transition at np 7792 in the mtDNA coding region, information about its geographic distribution is very scarce - so far it has been recognized in three individuals of European (Slovakia, England) and the southern Caucasus region ancestry (Figure S3). Coalescence age estimates for U5a1g is about 9 kya thus placing its origin to the Holocene.

The U5a1a’g cluster itself (based on HVS1 sequence data) is concentrated in populations of the Pontic-Caspian steppe, extending from Romania, Ukraine, southern Russia and northwestern Kazakhstan to the Ural Mountains. The highest frequencies of the U5a1a’g were reported in the Volga-Ural region (5.3%), in particular in Bashkirs (4.3%) and Tatars (3.9%) [75], although the frequency varies from ∼2.7% in Russians to ∼1.5% in populations of the northern Caucasus [64], [76]–[81]. It is worth mentioning that despite the low frequency of U5a1a’g haplotypes in Central Asian populations of Turkmens, Karakalpaks, Kazakhs and Uzbeks (∼1.5% according to the data of [82], some haplotypes were common between Karakalpaks (haplotype marked by mutation at np 16293), Turkmens (by mutation at np 64) and Iranians. So, it seems likely that the sub-cluster U5a1g or its founder has arrived to Iran from Eastern Europe/southern Ural via the Caspian Sea coastal route.

Another lineage potentially informative in revealing dispersals alongside the Eurasian steppe belt, extending from Manchuria to Europe, is haplogroup A4. Its rare sub-cluster A4h, with a specific HVS2 motif 97T-105-110del, revealed in Qashqais, has been observed earlier in Turkmens and Kazakhs [81], as well as in Buryats from southern Siberia [63]. Another A4 mtDNA lineage found in Qashqais, A4a1, has been registered previously in Central Asian Karakalpaks and Mongols, as well as in southern Siberian populations of Buryats and Altaians [63], [82]. It is noteworthy that another eastern Eurasian-specific mtDNA lineage, C5c, characteristic of some European populations (Poles, Russians, Ukrainians, Belarusians, Romanians), has been also found in the Iranian gene pool. Moreover, the complete mtDNA genome phylogeny points to Iranian rather than South Siberian origin of the European specific branch C5c1 dated to around 10 kya (Figure S3). As far as a significant influx of western Eurasian mtDNA lineages has been revealed in southern Siberian populations [63], the gene flow alongside the Eurasian steppe belt seems to be bi-directional.

Overall, this study provides a first extensive survey of complete mtDNA genome variation in Iran, useful for generating a more comprehensive history of the peoples of this region as well as for reconstructing ancient migration events. In addition, our results emphasize the importance of the Iranian Plateau as a source and recipient of gene flow between culturally, linguistically and genetically distinct populations.

## Supporting Information

Figure S1 Geographic location of sampling sites, with sample sizes given in parentheses.
(TIF)

Figure S2 **Maximum-parsimony phylogenetic tree of 352 Iranian complete mtDNA sequences, constructed using the program mtPhyl.** Numbers along links refer to substitutions scored relative to the rCRS [42]. Transversions are further specified; ins and del denote insertions and deletions of nucleotides, respectively; back mutations are underlined; symbol < denotes parallel mutation; heteroplasmies are labeled using the IUPAC code. For phylogeny reconstruction, the length variation in the poly-C stretches at nps 303-315 and 16184-16194 was not used. Polymorphism at np 16519 and A-C transversions at nps 16182 and 16183 were excluded. Iranian samples labeled as in Table S1. Established haplogroup labels are shown in black; blue are redefined and red are newly identified haplogroups in the present study.
(XLSX)

Figure S3 Maximum-parsimony phylogenetic trees of complete mtDNA sequences belonging to haplogroups U1, U3, U5a1g, U7, H13, HV12, N2a, W6, M5a, C5c, constructed using the program mtPhyl. Numbers along links refer to substitutions scored relative to rCRS [42]. Transversions are further specified; ins and del denote insertions and deletions of nucleotides, respectively; back mutations are underlined; symbol < denotes parallel mutation; heteroplasmies are labeled using the IUPAC code. Iranian samples labeled as in Table S1, for published data the accession number in indicated. The box containing the sample ID is color coded according to the geographic origin of the sample. Coalescence time estimates expressed in kya are shown along links next to clade labels and were calculated by computing the averaged distance (r) and the standard error (s) [45], [46]. Calculations were obtained using the entire mtDNA genomes but excluding the length variation in the poly-C stretches at nps 303-315 and 16184-16194 and hot spot mutations such as 16182C, 16183C, and 16519. Values of mutation rates based on mtDNA complete genome variability data and synonymous substitutions [47] were used. Established haplogroup labels are shown in black; blue are redefined and red are newly identified haplogroups in the present study.
(XLS)

Figure S4 BSP indicating the median of the hypothetical effective population size through time based on complete mtDNA genome data from the mtDNA haplogroups U7 and H13. Maximum time (x axis) corresponds to the median posterior estimate of the genealogy root-height.
(PDF)

Table S1 Detailed description of Iranian samples studied. Established haplogroup labels are shown in black; blue are redefined and red are newly identified haplogroups in the present study.
(XLSX)

Table S2 Diversity indices and neutrality test for Iranian, Caucasian, Anatolian and some European populations based on complete mtDNA sequences.
(XLS)

Table S3 Pairwise Fst values between ten populations from the Caucasus, Iran, Anatolia, and Europe.
(XLS)

Table S4 AMOVA results for populations of Iran, Caucasus, Anatolia, and Europe based on complete mtDNA variation.
(XLS)
