# Supplementary figures and images for: Complete Mitochondrial DNA Diversity in Iranians
Source: PLoS One. 2013 Nov 14;8(11):e80673. doi: 10.1371/journal.pone.0080673 (PMC3828245; doi:10.1371/journal.pone.0080673)

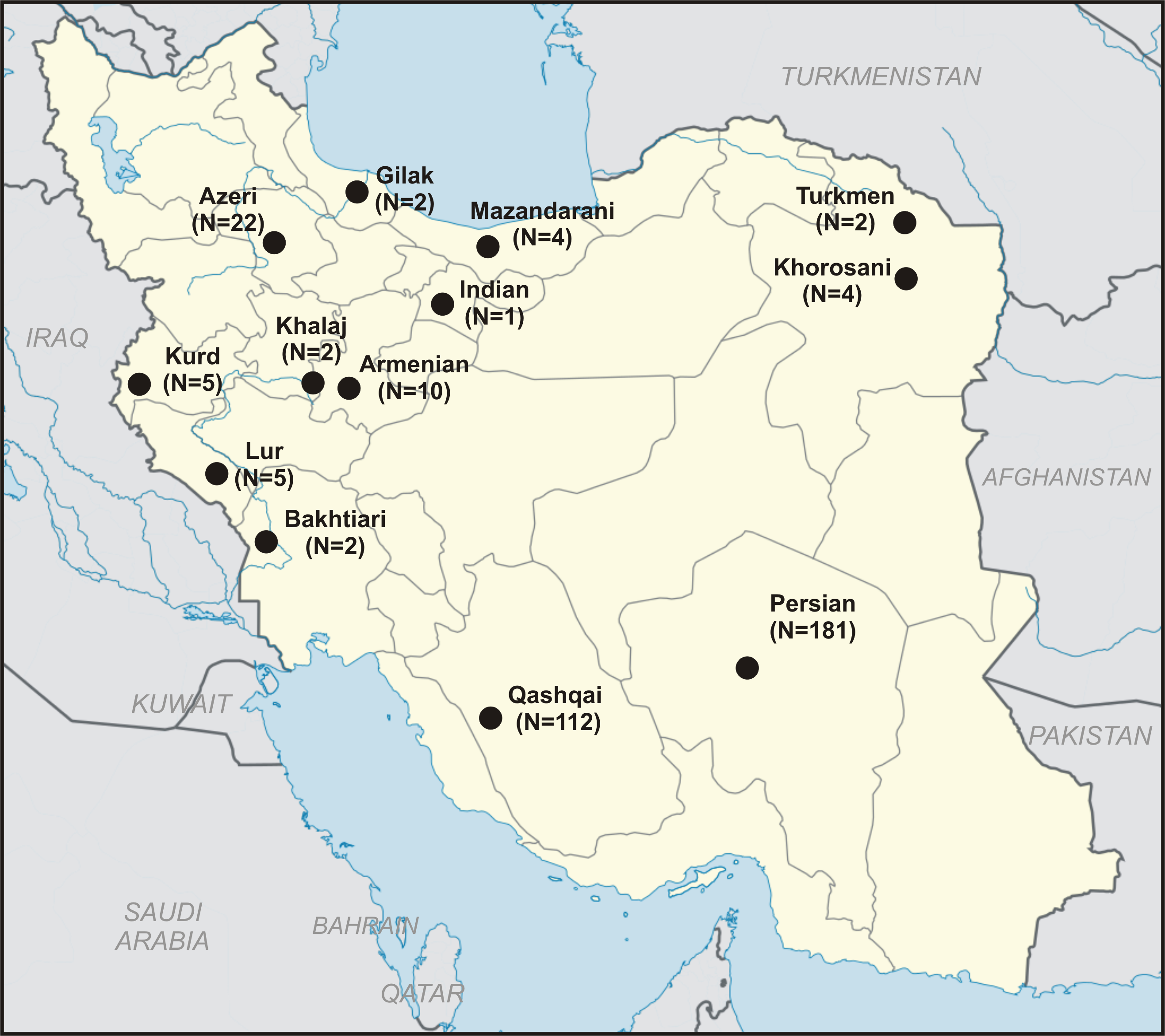

Supplement: Figure S1 — Geographic location of sampling sites, with sample sizes given in parentheses. (TIF) [file pone.0080673.s001.tif]
