# Supplementary material for: Complete Mitochondrial DNA Diversity in Iranians
Source: PLoS One. 2013 Nov 14;8(11):e80673. doi: 10.1371/journal.pone.0080673 (PMC3828245; doi:10.1371/journal.pone.0080673)
Supplement: Figure S4 — BSP indicating the median of the hypothetical effective population size through time based on complete mtDNA genome data from the mtDNA haplogroups U7 and H13. Maximum time (x axis) corresponds to the median posterior estimate of the genealogy root-height. (PDF) [file pone.0080673.s004.pdf]

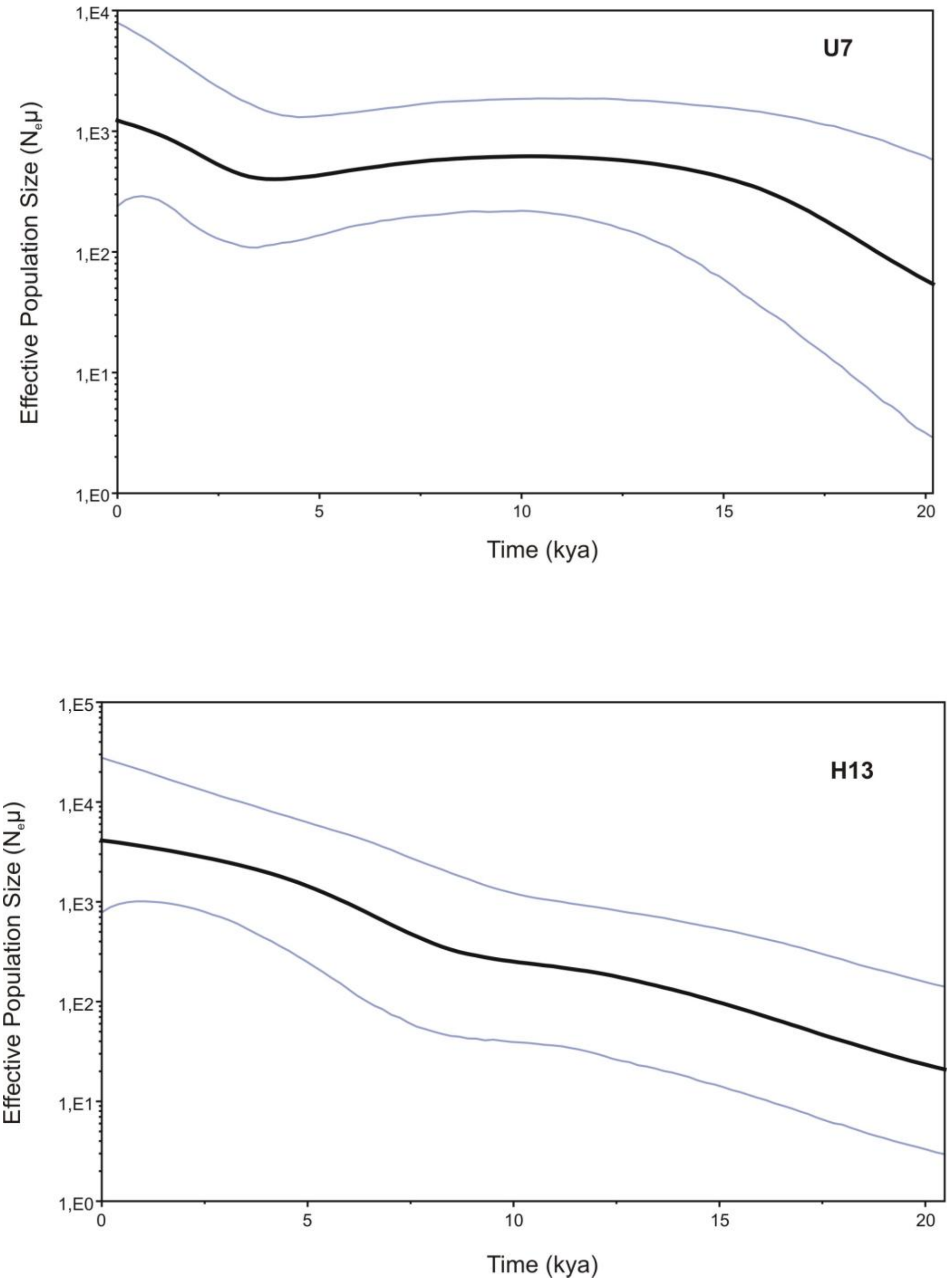

Figure S3. BSP indicating the median of the hypothetical effective population size through time based on complete mtDNA genome data from the mtDNA haplogroups U7 and H13. Maximum time (x axis) corresponds to the median posterior estimate of the genealogy root-height.
